# An Emerging Preventive Mental Health Care Strategy: The Neurobiological and Functional Basis of Positive Psychological Traits

**DOI:** 10.3389/fpsyg.2021.728797

**Published:** 2021-10-22

**Authors:** Ashten R. Duncan, Grant Daugherty, Gabrielle Carmichael

**Affiliations:** ^1^Department of Family and Community Medicine, University of New Mexico, Albuquerque, NM, United States=; ^2^OU-TU School of Community Medicine, University of Oklahoma, Tulsa, OK, United States; ^3^OU College of Medicine, University of Oklahoma, Oklahoma City, OK, United States

**Keywords:** preventive mental health care, neurobiology, positive psychology, hope theory, psychometrics, clinical settings

## Abstract

Even with the expanding burden of the COVID-19 pandemic on mental health, our approach to mental health care remains largely reactive rather than preventive. This trend is problematic because the majority of outpatient visits to primary care providers across the country is related to unmet mental health needs. Positive psychology has the potential to address these issues within mental health care and provide primary care providers with strategies to serve their patients more effectively. Positive psychology has many frameworks like hope, which can be measured using simple questionnaires in the waiting room. Moreover, there is a growing body of neurobiological evidence that lends credence to positive psychology concepts in the context of differential neuronal activation patterns. Many positive psychological instruments not only have high construct validity but also have connections to observable neurobiological differences tied to differences in psychosocial functioning. Despite the current evidence, we still need robust research that explores if such psychometric measurements and related interventions lead to clinically significant and favorable health outcomes in patients outside of controlled environments.

## Introduction

The COVID-19 pandemic has resulted in devastating worldwide consequences for health networks and patients. Along with economic and medical repercussions, there has also been an unprecedented rise in the prevalence of mental illnesses (Panchal et al., 2021). Recent data show the number of adults reporting anxiety or depressive symptoms has risen from about 10 to 40% of the population (Panchal et al., 2021). Even with the expanding burden of the pandemic on mental health, our approach to mental health care remains largely reactive rather than preventive. This emphasis on treating active psychopathology means that we are likely missing key opportunities to mitigate the effect of social isolation and disease-related distress in the USA (Panchal et al., 2021).

Mental health care still faces stigmatization from much of the public, ranging from hesitancy to distrust (Parcesepe and Cabassa, 2012). This stigma largely stems from the fact that many people do not see mental illnesses as legitimate as somatic dysfunctions (Parcesepe and Cabassa, 2012). Unfortunately, these perceptions of mental health create a challenge for both patients and primary care providers (PCPs) given that approximately 70% of outpatient visits relate to unmet mental health needs (Hunter et al., 2017). Among PCPs, a lack of clinical tools needed to provide preventive mental health services frequently hinders efforts to find intervention points before a decline in function appears (Clatney et al., 2008). Therefore, PCPs need tools that are readily implementable in busy practices and that lead to clinically appreciable changes in well-being.

This article seeks to highlight the potential of positive psychology to address these issues within mental health care and provide PCPs with strategies to serve their patients more effectively. Positive psychology has many frameworks like hope, which can be measured using a simple questionnaire in the waiting room. Many of these positive psychological instruments not only have high construct validity but also have connections to observable neurobiological differences. Additionally, these scales are generally available for free, provide a unique perspective into the spectrum of mental well-being, and are actionable in a variety of patient populations. While these scales are widely available, they are not currently widely implemented. Therefore, we argue positive psychology has significant, untapped clinical utility for preventive mental health care in primary care settings. Further research into how these variables affect clinical outcomes will likely be both exciting and necessary.

## Mental Health Care and the Role of Positive Psychology

One concept that has gained traction in the realm of preventive health care is salutogenesis, which is the idea of promoting and improving health even in the absence of disease (Bauer et al., 2020). While supportive psychotherapy and developing coping skills have such salutogenic utility, they are not the only effective means of attaining mental well-being and not necessarily what every patient needs long-term (Bauer et al., 2020). In fact, many patients would likely benefit more from developing neuropsychoplasticity and protective factors gradually during routine clinical encounters, which may be key to preventing new or worsening psychopathology (Jeste et al., 2015; Bauer et al., 2020). This movement in clinical mental health care toward salutogenesis espouses principles of the positive psychology movement, which Martin Seligman championed in the 1990s (Jeste et al., 2015).

Since the 1990s, Martin Seligman and his colleagues have conducted a plethora of studies ranging from animal model experiments to integrated biopsychological studies of humans to demonstrate the validity and modifiability of positive psychological traits and states and to challenge the old paradigm wherein mental well-being was the default and psychopathology was the breakdown in this baseline well-being. As a powerful example of this paradigm shift, Seligman and others explored the concept of learned helplessness, which described animals’ natural tendencies to learn defeatist behaviors and lose their capacity for adaptability in the face of adversity (Maier and Seligman, 2016). It is now clear from neurobiological research that helplessness and passivity are the default decreases in adaptive brain functions secondary to prolonged aversive experiences and that learned control is the adaptive, modifiable response (Maier and Seligman, 2016). Seligman has further elucidated this principle with his conceptualization of the “hope circuit,” which shows how the medial prefrontal cortex develops as we learn control and subsequently inhibits the dorsal raphe nucleus to prevent dispositional helplessness (Seligman, 2019). This evidence shows how leveraging positive psychological traits and states can enhance well-being beyond simply reacting to declines in mental health.

Positive psychology interventions (PPIs) likely have clinical importance in primary care settings where patients’ overall health is a major priority (Park et al., 2014). A large-scale meta-analysis of PPIs demonstrated significant improvements in well-being, depression, and anxiety measures from pre- to post-intervention (Chakhssi et al., 2018). The PPI literature shows that these theory-grounded strategies are effective across a variety of settings and delivery formats, which underscores the flexibility of these approaches (Chakhssi et al., 2018). This evidence strongly suggests that PPIs can play a unique role in the primary prevention of common psychopathological conditions since they have beneficial effects on mental health outcomes and do not need to be delivered in a strictly prescribed manner (Chakhssi et al., 2018). Moreover, current research also signals the potential utility of PPIs outside of psychiatrists’ and psychologists’ offices, such as in primary care settings where mental health issues are so common (Wittchen et al., 2003; Hunter et al., 2017).

Therefore, better understanding the role of positive psychology in mental health care may pave the way for novel, community-level health promotion strategies that could decrease both the incidence and severity of common psychopathological conditions. Emerging evidence supports how changes in positive psychological traits are associated with structural brain changes. Moreover, these changes are known to have associations with adaptive mental functions. Therefore, it is necessary to examine the connection between positive psychology and neurobiology.

## The Brain Structure–Function Relationship and Hope

Strong evidence supports a brain structure–function relationship wherein structure determines function (Batista-García-Ramó and Fernández-Verdecia, 2018). This is analogous to antecedent anatomical disruptions changing functional outcomes in other organ systems (Murphy et al., 2018). Neuroanatomical research continues to elucidate the connections between brain structures and their functions, opening new avenues for improving health care practices (Batista-García-Ramó and Fernández-Verdecia, 2018; Murphy et al., 2018). This work is in the same vein as Seligman’s hope circuit research, which adds to the consistency and specificity of the evidence in this area (Seligman, 2019). While this field of scientific research is still relatively new, discoveries in this area are leading to important breakthroughs that allow us to treat human suffering more effectively and promote psychological well-being (Mayberg et al., 2005). In light of these advances in neuroimaging research, increasing understanding of the brain structure–function relationship may encourage a paradigm shift in the public’s perception of and approach to mental illness.

Unlike other complex organs like kidneys, a central feature of this paradigm shift is the recognition that the brain is amenable to distinct types of non-physical therapies. For example, cognitive behavioral therapy has demonstrable benefits in altering the neuroanatomical activation patterns involved in the remission of common mental illnesses like major depression, such as increased glucose metabolism in several prefrontal cortical areas (Beauregard, 2014). An increasing number of studies indicates that PPIs can change people’s hormonal, immunological, and neurological activity and produce differential neuronal firing patterns in the brain (González-Díaz et al., 2017).

Positive psychology consists of manifold concepts, including flourishing, hope, gratitude, and creativity (Hausler et al., 2017). On account of its direct connections to clinical care, it is worthwhile to examine hope more closely as a well-studied example of positive psychology (Duncan et al., 2020). Hope represents an individual’s cognitive appraisal of internal and external resources related to pursuing a desired goal, such as motivation and social support (Snyder et al., 1991; Duncan et al., 2020). In effect, dispositional hope is a measure of a person’s baseline capacity for goal setting, perseverance when faced with goal-related barriers, and adaptability during goal pursuit (e.g., setting subgoals and alternative goals; Snyder et al., 1991; Duncan et al., 2020). Researchers have operationalized hope by developing instruments like the Adult Hope Scale (AHS) that assess approach versus avoidance behaviors through goal-oriented questions (Snyder et al., 1991). A recent synthesis of research at the intersection of hope and mental illness revealed that hope plays an integral role in the recovery process and can be increased despite the neuropsychiatric effects of many mental illnesses (Schrank et al., 2012). The current evidence sufficiently shows that interventions resulting in dispositional hope increases can be tailored to the individual, which suggests potentially high therapeutic utility (Snyder et al., 1991; Schrank et al., 2012; Duncan et al., 2020).

Recent neuroimaging studies exploring changes in dispositional hope have shown how hope may change the brain. One study using resting-state functional magnetic resonance imaging revealed that higher dispositional hope was positively correlated with more spontaneous neuronal firing in the bilateral medial orbitofrontal cortices and negatively correlated with anxiety severity (Wang et al., 2017). In a follow-up study, higher hope was associated with greater gray matter volume of the left supplementary motor area, more satisfaction with life, and higher hedonic balance ratings (Wang et al., 2020). All of these findings suggest that interventions that change hope also produce changes in different types of cortical activity connected to adaptive functions like emotional regulation, goal setting, and impulse control (Batista-García-Ramó and Fernández-Verdecia, 2018). Therefore, measuring dispositional hope appears to give some insight into patients’ neurobiology without the need for impractical neuroimaging.

## Implications of Measuring Hope for Health Care

Using psychometric scales like the AHS as assessment tools requires few resources beyond informing providers about how to interpret the scores (Snyder et al., 1991). Having the AHS in wellness toolkits could potentially heighten providers’ awareness of opportunities to support patients before a mental health crisis or disorder develops. These scales and their accompanying intervention strategies have salutogenic and preventive mental health care utility in a wide variety of settings, which may be able to lessen the tremendous burden on the existing mental health infrastructure (Wittchen et al., 2003; Clatney et al., 2008; Schrank et al., 2012). Additionally, providers could tailor their choice of intervention to what would benefit the patient most. A personalized psychological intervention would offer greater treatment efficacy, and its foundation in observable neurostructural changes may also garner trust and acceptance from those who are hesitant to accept mental health care.

By demonstrating the specific clinical applications and limitations of measuring psychological traits like hope and appropriately implementing related PPIs, psychometric scales grounded in positive psychological theories could gain the same confidence as hemoglobin A1c or blood pressure screenings among patients and mental health care providers. These psychometric tools could be employed in the same manner as a review-of-systems checklist while patients are in a waiting room to identify opportunities to help them. By applying these tools universally, we could lessen the stigma and burden surrounding mental health conversations. Providers would no longer have to guess which patients require intervention, and patients would no longer feel singled out. Moreover, we could identify more points of intervention earlier and thereby prevent suffering. Just as physicians and other providers inquire about diet, exercise, and sleep habits because of their relevance to health, they could similarly normalize questions about goal setting to know if and when a patient is ready to set further health-related goals.

## Knowledge Gaps and Future Research Directions

We are developing a better understanding of the connection between neurobiology and neuropsychological function. Mapping hope to differential neurostructural activity is just one of multiple examples of this progress. However, much remains to be discovered in connecting biological factors to a person’s behavioral tendencies and psychological well-being. Given what we know about the role of hope and other positive psychological traits on brain activity, it would likely be fruitful to continue expanding on the extant basic science research. For applied clinical research, studying the use of these psychometric measurements in the context of complex health-goal- and risk-related behaviors like non-adherence, lifestyle modification adoption, and cessation of substance use could produce substantive and powerful practice recommendations. This step is essential for making all of this relevant to clinical practice and increasing the uptake of these theory-supported concepts.

Specifically, psychometric testing could be administered while patients are waiting, minimizing disruptions to standard workflows. Administration of PPIs could be handled through a referral system wherein patients with low psychometric scores could be offered a chance to participate in individual or group treatments with a trained facilitator. In conjunction with physical and mental health data, the results of such interventions could be tracked over time as the patient continues to use services in order to detect long-term effects. By implementing such a system, researchers could study the impacts of positive psychology interventions and their clinical significance.

## Conclusion

Hope and the broader field of positive psychology have a unique potential to improve mental well-being in novel ways. Positive psychological concepts like hope show robust construct validity and are appreciable on a neurobiological level. Researchers have developed widely available psychometric instruments and have used these tools to show that PPIs effectively improve these traits. To generalize these findings further, we need research that explores if these psychometric measurements and related PPIs lead to clinically significant and favorable health outcomes in patients outside of controlled environments. Such research could produce sufficiently high external validity to warrant using these tools in larger community and primary care settings.

As our understanding of brains as dynamic, highly sensitive organs grows, we need to counsel our patients that maintaining mental health is a process that demands the same attention as diets and exercise habits. A key part of encouraging this perspective involves improving the balance between proactive and reactive mental health services. This will entail expanding the clinical evidence in order to earn the confidence of PCPs, who could then speak to their patients about the benefits of the positive psychological frameworks. Such a widespread understanding would encourage those with mental health concerns to seek care when needed and increase awareness of the importance of preventive mental health care.

## Data Availability Statement

This article reports on the findings from other researchers’ original studies. Further inquiries can be directed to the corresponding author.

## Author Contributions

AD, GD, and GC contributed to the literature search, manuscript design, writing, editing, and topic selection involved in preparing this article. AD led the research team and proposed the initial concept underlying this work. All authors contributed to the article and approved the submitted version.

## Conflict of Interest

The authors declare that the research was conducted in the absence of any commercial or financial relationships that could be construed as a potential conflict of interest.

## Publisher’s Note

All claims expressed in this article are solely those of the authors and do not necessarily represent those of their affiliated organizations, or those of the publisher, the editors and the reviewers. Any product that may be evaluated in this article, or claim that may be made by its manufacturer, is not guaranteed or endorsed by the publisher.
